# Practical Remarks upon Ipecacuanha, with a Formula for a More Uniform and Efficient Preparation of the Syrup, Than That Laid down in the Dispensatories

**Published:** 1850-10

**Authors:** Edward Jenner Coxe

**Affiliations:** New Orleans


### MATERIA MEDICA AND THERAPEUTICS.

*Practical Remarks upon Ipecacuanha, with a formula for a more uniform and efficient preparation of the syrup, than that laid down in the Dispensatories.* By Edward Jenner Coxe, M. D., New Orleans. —Before noticing the main object of these remarks, it may prove neither uninteresting nor unprofitable to direct attention to some of those diseases in which this medicine, or some of its preparations and combinations, may be employed. The value and efficacy of ipecacuanha, as an emetic or expectorant in many affections of the respiratory organs, more particularly of children, are too generally conceded and acted upon to require an extended notice.

In dysentery, ipecacuanha has been and continues to be much used.

By Mosely who held it in high repute, ipecacuanha was given in doses of half a drachm to two scruples, and by the late Professor B. S. Barton, it was regarded as almost a specific, particularly in cases of a typhoid character. In chronic diarrhœa, small doses of the powder repeated several times a day, either alone, or preferably in conjunction with opium or Dovers powder, will be found of great value and frequently, with strict attention to a proper regimen, will succeed in curing many most unpromising cases.

In these last cases, when dependent upon, or connected with derangement of the biliary secretion, additional power will be given to the above by uniting with them two or three grains of blue mass to be repeated every night for three or four nights, and subsequently every third or fourth night as long as may be deemed requisite or advisable for the individual case. In hemorrhage from the lungs, or uterus, small doses of ipecacuanha, combined with sugar of lead and opium are used with decided benefit.

In hemorrhage from the stomach, large doses of ipecacuanha have been strongly recommended, more particularly by Dr. Condie who has published some valuable practical remarks upon the subject.

In the early stages of the bowel affections of children, no less than in adults, an emetic of ipecacuanha, will often succeed in arresting the progress of the disease, and rarely fail to prove beneficial.

Combining from one fourth to half a grain of ipecac. with a minute portion of opium, and two or three grains of blue mass, the alterative properties of this last are materially enhanced, and will be found of great benefit in most of the mild cases of biliary and bowel derangements so prevalent in this region, at different seasons of the year.

With the exception of that sudden and often fatal disease, croup or hives, there are perhaps none of the pectoral diseases of children, in which the syrup of ipecacuanha may not be resorted to with advantage; but in croup, no little experience, and an almost uniform success in its treatment, authorize the confident belief, that we possess no one remedy or combination of remedies comparable or equal to the well known Coxe's hive syrup, provided it be properly prepared. Dr. Good has remarked, that the ipecacuans concur in operating very generally upon the skin, at the same time that they excite the stomach, increasing in a slight degree the discharge of mucus from the lungs, and adding a little to the peristaltic motion of the bowels, while the antimonials act more violently upon the stomach, bowels and skin, but less upon the mucous secernents.

To recur to the syrup of ipecacuanha, I may remark, that being the United States Dispensatory attended with unnecessary trouble, and, without constant care, great probability of a want of uniformity in the preparation, I adopted, after many trials, the following formula, which can be depended upon at the bed side, and which has been found to keep well in this climate.

| | |
|---|---|
| R.—Ipecacuan. Rad. Contus. | ℥iv. |
| Aqua | O.ij. |
| Sp. Vin. Rect. | ℥x. |
| Sacch. Alb. | lbs. iij. |

Macerate the bruised ipecac in one pint of boiling water for 12 hours, then add the remainder of the water and alcohol, and continue the maceration for five or six days. Place the whole in a small displacement apparatus, returning the fluid that passes until it becomes perfectly clear, and then continue to pour a small quantity of water occasionally upon the surface, until two pints and ten ounces by measure shall have passed. Now add the sugar, and with a gentle heat, evaporate until the syrup shall be of a proper consistence, readily ascertained by occasionally taking out a small portion and allowing it to cool. When of a proper consistence, pass it through a small quantity of fine tow placed in the tube of a funnel to render the syrup clear and transparent. Three pints and ten ounces of syrup is the quantity obtained, and is in point of strength nearly double of that prepared by the usual formula, which I consider an additional recommendation.

*N. O. Med. and Surg. Jour.*

